# Pedigree-based study to identify *GOLGB1* as a risk gene for bipolar disorder

**DOI:** 10.1038/s41398-022-02163-x

**Published:** 2022-09-17

**Authors:** Fa-rong Liu, Yunqiang Zhou, Yong Wang, Ling-ling Huang, Xian Zhang, Hong Luo, Su-ying Wu, Hai-yan Lyu, Li-huan Huang, Huaxi Xu, Yun-wu Zhang

**Affiliations:** 1 Xiamen Key Laboratory of Brain Center, The First Affiliated Hospital of Xiamen University, and Fujian Provincial Key Laboratory of Neurodegenerative Disease and Aging Research, Institute of Neuroscience, School of Medicine, Xiamen University, Xiamen, Fujian 361102 China; 2 Xiamen City Xianyue Hospital, Xiamen, Fujian 361012 China; 3 The Third Clinical Medical College, Fujian Medical University, Fuzhou, Fujian 350122 China

**Keywords:** Clinical genetics, Molecular neuroscience, Bipolar disorder

## Abstract

Bipolar disorder (BD) is a complex psychiatric disorder with strong heritability. Identification of new BD risk genes will help determine the mechanism underlying disease pathogenesis. In the present study, we carried out whole genome sequencing for a Chinese BD family with three affected members and three unaffected members, and identified multiple candidate causal variations, including a frameshift mutation in the *GOLGB1* gene. Since a *GOLGB1* missense mutation was also found in another BD pedigree, we carried out functional studies by downregulating *Golgb1* expression in the brain of neonatal mice. *Golgb1* deficiency had no effect on anxiety, memory, and social behaviors in young adult mice. However, we found that young adult mice with *Golgb1* deficiency exhibited elevated locomotor activity and decreased depressive behaviors in the tail suspension test and the sucrose preference test, but increased depressive behaviors in the forced swim test, resembling the dual character of BD patients with both mania and depression. Moreover, *Golgb1* downregulation reduced PSD93 levels and Akt phosphorylation in the brain. Together, our results indicate that *GOLGB1* is a strong BD risk gene candidate whose deficiency may result in BD phenotypes possibly through affecting PSD93 and PI3K/Akt signaling.

## Introduction

Bipolar disorder (BP) is a complicated neuropsychiatric disorder that shows mood changes between mania and depression. BD can be classified into type I and type II, for which type I BD is characterized by the presence of a syndromal, manic episode, and type II BD is characterized by the presence of a syndromal, hypomanic episode, and a major depressive episode [1, 2].

BD affects nearly 2% of the world’s adult population and has a heritability of about 60–85% [3–5]. Genetic studies, such as genetic linkage studies, candidate gene studies, and genome-wide association studies (GWAS), have identified multiple BD susceptibility loci [6–9]. Although some of them, such as *BDNF*, *ANK3*, and *CACNA1C*, may be common susceptibility loci for BD, most identified loci are rare, implicating a polygenic contribution of common and rare variations to BD susceptibility [3, 10]. Since, so far, identified loci only explain a portion of BD occurrence, further investigation in affected pedigrees may identify additional genetic loci that contribute to BD susceptibility.

In the present study, we carried out whole genome sequencing (WGS) to identify rare susceptibility variations for BD in a Chinese BD pedigree. We identified multiple variations, including a nonsense mutation in the *GOLGB1* gene in affected family members but not in unaffected family members. The *GOLGB1* gene encodes GOLGB1/Giantin, a protein belonging to the golgin family members that reside in the Golgi stack and modulate vesicle trafficking [11]. Although GOLGB1 has been proposed to regulate protein glycosylation [12], ciliogenesis [13, 14], and osteogenesis and/or chondrogenesis [12, 15], the exact function of GOLGB1 has yet to be further elucidated. Since a missense mutation in the *GOLGB1* gene was also found in affected but not unaffected members in another BD pedigree [4], we studied mice with reduced *Golgb1* expression in the brain. The results showed that *Golgb1* deficiency resulted in some behavior abnormalities resembling those found in BD patients, suggesting that GOLGB1 dysregulation may contribute to certain BD phenotypes.

## Results

**Fig. 1:**
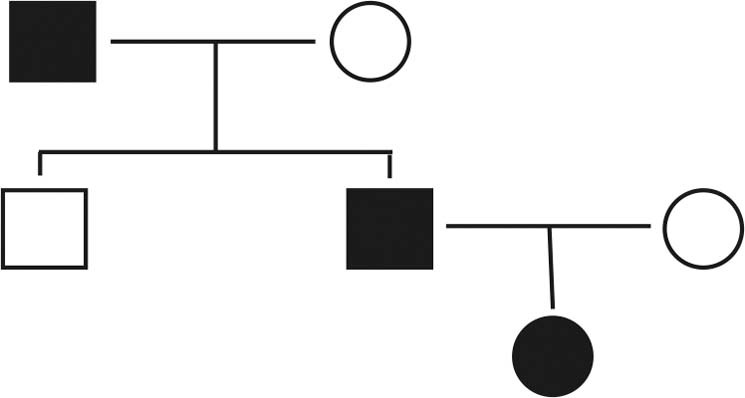
Pedigree of a Chinese family with three members diagnosed with BD. In this pedigree, family relationships are indicated by lines. Squares indicate males. Circles indicate females. Filled symbols indicate affected members (with BD). Unfilled symbols indicate unaffected/healthy members.

We carried out WGS for three affected and three unaffected members in a Chinese BD family (Fig. 1). WGS analysis revealed a total of 5,472,225 single-nucleotide variations (SNVs) and 1,251,821 small insertions and deletions (INDELs) present in cases and controls combined. Among them, we filtered 940 SNVs and 142 INDELs that were potentially deleterious. Given the inheritance pattern of the pedigree, autosomal dominant genetic modifiers seem to be responsible for disease pathogenesis in this family. Therefore, from filtered variations, we further screened variations that were heterozygous in all three BD patients but not mutated in healthy members within this family as candidate causal variations. We found 23 SNVs and 3 INDELs, each of which located in one individual gene, as candidate causal variations (Table 1).

**Table 1:** Potential gene variations associated with bipolar disorder identified by WGS.

Potential casual genes	Mutation types	Transcript	Exon	Coding	Protein	Association with BD in other studies
*RAB3GAP1*	SNV-missense	NM_001172435	25	C2821G	P941A	-
*GPD2*	SNV-missense	NM_001083112	9	A1096G	I366V	-
*CCR4*	SNV-missense	NM_005508	2	G424A	A142T	-
*SCN10A*	SNV-missense	NM_006514	13	C2015T	T672I	-
*GOLIM4*	SNV-missense	NM_014498	4	A334G	S112G	-
*ADH5*	SNV-missense	NM_000671	4	C328T	L110F	-
*PELO*	SNV-missense	NM_015946	2	A716G	K239R	-
*TTC37*	SNV-missense	NM_014639	11	G829A	G277S	-
*NEU1*	SNV-missense	NM_000434	4	C640T	R214C	[16]
*MDN1*	SNV-missense	NM_014611	63	C10551A	D3517E	-
*SPAG1*	SNV-missense	NM_172218	9	C844T	R282C	-
*APTX*	SNV-missense	NM_175073	9	C952A	R318S	-
*AKNA*	SNV-missense	NM_030767	3	G577C	V193L	-
*COG6*	SNV-missense	NM_020751	12	A1145G	K382R	-
*MAP3K9*	SNV-missense	NM_033141	13	A2936G	N979S	-
*YLPM1*	SNV-missense	NM_019589	5	T3638C	M1213T	-
PKD1L2*	SNV-unknown	unknown	16	unknown	unknown	-
*CCDC40*	SNV-missense	NM_017950	20	C3355T	P1119S	-
*TBCD*	SNV-missense	NM_005993	20	C1810T	P604S	-
*CYB5A*	SNV-missense	NM_148923	1	G25T	V9L	-
*ELANE*	SNV-missense	NM_001972	2	C100T	R34W	-
*DOT1L*	SNV-missense	NM_032482	20	T2250G	C750W	-
*TMPRSS15*	SNV-missense	NM_002772	4	G428T	G143V	-
*GOLGB1*	INDEL-frameshift deletion	NM_004487	15	8743delC	H2915fs	[4]
*EYS*	INDEL-nonframeshift deletion	NM_001292009	19	2953_2961del	TDG985_987del	-
*TBC1D16*	INDEL-frameshift deletion	NM_019020	5	1015delC	H339fs	[17]

^*^The representative *PKD1L2* transcript (NM_052892) is present in some human individuals but absent from the reference genome. Therefore, the effects of the identified SNV on its coding and protein sequences are unknown.

For the 26 genes carrying SNVs or INDELS potentially associated with BD, we performed PubMed (https://pubmed.ncbi.nlm.nih.gov/) literature research using each gene name and “bipolar” as keywords (as of 08/11/2022). We found that *NEU1* [16], *TBC1D16* [17], and *GOLGB1* [4] have previously been linked to BD (Table 1). Interestingly, one previous study identified a missense mutation in the *GOLGB1* gene (c.983 T > C, p.V328A, in exon 9, NM_004487) in affected members but not unaffected members in a Caucasian BD family [4]. Here we identified a frameshift INDEL in the *GOLGB1* gene (c.8743delC, p.H2915fs, in exon 15, NM_004487) in affected members but not in unaffected members in this Chinese BD family. Therefore, we targeted *GOLGB1* for further analysis.

The human *GOLGB1* gene is a big gene on chromosome 3 and has 27 exons with multiple splicing variants. Focusing on the two mutations potentially associated with BD, we sequenced entire exons 9 and 15 of *GOLGB1* in 182 sporadic BD patients and 146 controls. However, we did not identify the two or other mutations in the studied subjects, suggesting that the two mutations are rare.

**Fig. 2:**
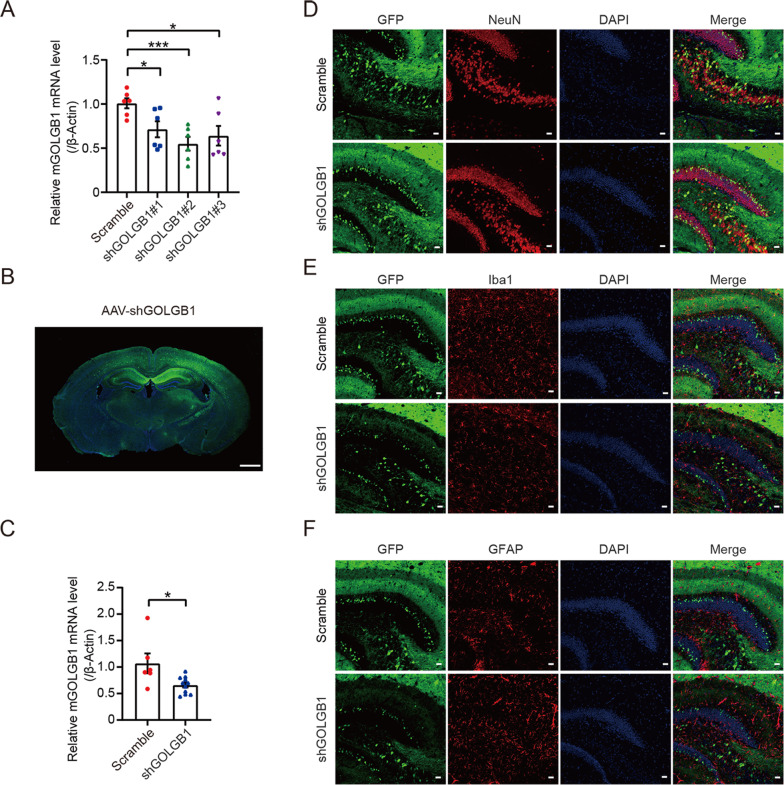
Downregulation efficiency and expression localization of AAV-GOLGB1 shRNAs. **A** Mouse primary neurons were infected with AAVs expressing different mouse *Golgb1* shRNAs (shGOLBG1 #1, #2, and #3) or a scrambled control shRNA. The mRNA levels of mouse *Golgb1* were determined by qRT-PCR and normalized to those of β-actin for comparison. *n* = 6; *p < 0.05, ***p < 0.001; two-tailed Student’s *t*-test. **B** Representative image of the GFP expression (in green) in mice injected with AAVs. The nuclei were counterstained with DAPI (in blue). Scale bar: 1 mm. **C** RNAs were extracted from hippocampal tissues of mice injected with AAVs expressing shGOLGB1 or a scrambled control shRNA. The mRNA levels of mouse *Golgb1* were determined by qRT-PCR and normalized to those of β-actin for comparison. *n* = 11 for shGOLGB1 mice, *n* = 6 for scrambled control mice; *p < 0.05; two-tailed Student’s *t*-test. **D–F** The brain of mice injected with GFP (in green)-containing AAVs expressing shGOLGB1 or a scrambled control shRNA were sectioned. Brain sections were immunostained with the neuron marker NeuN (**D**, in red), the microglia marker Iba1 (**E**, in red), and the astrocyte marker GFAP (**F**, in red), and counterstained with DAPI (in blue). Scale bars: 30 μm. The hippocampal regions were observed under a confocal microscope.

Because our identified *GOLGB1* INDEL leads to a predicted early stop of the coding sequence and truncation of the protein, we also studied whether GOLGB1 deficiency causes BD-like phenotypes in animals. We first packaged AAVs that express different mouse *Golgb1* shRNAs and tested their efficiency in downregulating *Golgb1* in mouse primary neurons. We found that all three tested *Golgb1* shRNAs significantly reduced mouse *Golgb1* mRNA levels, with shGOLGB1 #2 showing the most effect on reducing *Golgb1* compared to the other two (Fig. 2A).

Next, we delivered AAVs expressing shGOLGB1 #2 or scrambled controls into the brain of P0 mice via bilateral intracerebroventricular (i.c.v.) injection. GFP fluorescence represented the localization of AAVs and indicated that AAVs infected mostly hippocampal and cortical regions (Fig. 2B). We confirmed that shGOLGB1 #2 expression significantly reduced *Golgb1* expression in mouse hippocampal tissues (Fig. 2C). Furthermore, GFP was found to colocalize with the neuron marker NeuN (Fig. 2D) but not with the microglia marker Iba1 (Fig. 2E) or the astrocyte marker GFAP (Fig. 2F), suggesting that AAVs mostly infected neurons, i.e., *Golgb1* was mostly downregulated in neurons.

**Fig. 3:**
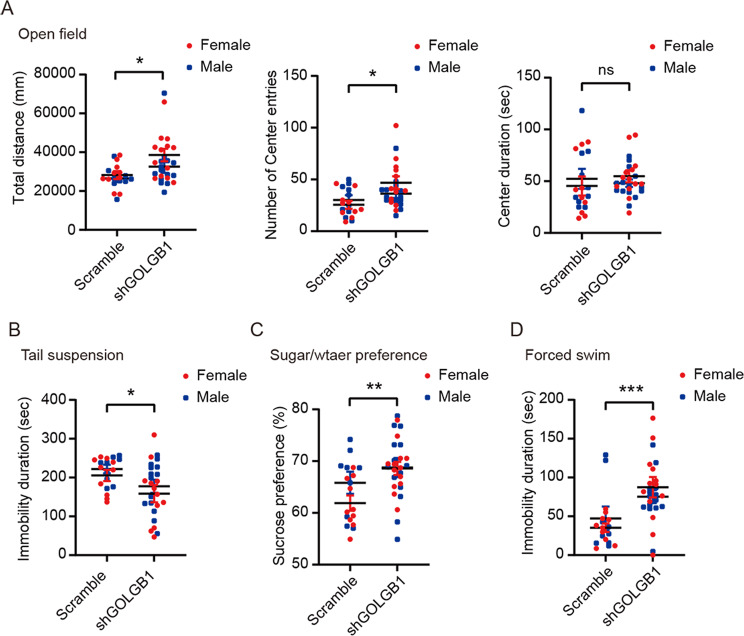
Downregulation of *Golgb1* causes BD-like behaviors in mice. Mice of both sexes injected with AAVs expressing shGOLGB1 or a scrambled control shRNA were subjected to behavioral tests at 2 months of age. **A** In the open field test, mice were studied for their total travel distance, their numbers of center entries, and their duration time in the center. **B** In the tail suspension test, mice were studied for their immobility duration during the tail suspension. **C** In the 2-bottle choice sucrose preference test, mice were studied for their preference for sucrose. **D** In the forced swim test, mice were studied for their immobility duration in water. *n* = 27 (13 females and 14 males) for shGOLGB1 mice, *n* = 19 (ten females and nine males) for scrambled control mice; ns not significant, *p < 0.05, **p < 0.01, ***p < 0.001; two-tailed Student’s *t*-test.

We next investigated whether *Golgb1* knockdown (KD) affects mouse behaviors. Two months after AAV infection, both male and female mice were subjected to various behavioral tests. Since both sexes are affected in this BD family, we combined data from mice with both sexes for comparisons. In the open field test, we found that *Golgb1* KD mice were more active than control mice, as they exhibited significantly increased total travel distance and numbers of center entries (Fig. 3A). In the tail suspension test, we found that *Golgb1* KD mice had less immobility time than control mice (Fig. 3B), implying a decrease of depression in *Golgb1* KD mice. Consistently, *Golgb1* KD mice had increased sucrose preference indicative of decreased depression compared control mice in the two-bottle choice sucrose preference test (Fig. 3C). Surprisingly, in the forced swim test, *Golgb1* KD mice showed significantly elevated immobility time, implying increased depression compared to controls (Fig. 3D). Together, these behaviors may resemble those found in BD patients, who exhibit mood change between mania and depression.

**Fig. 4:**
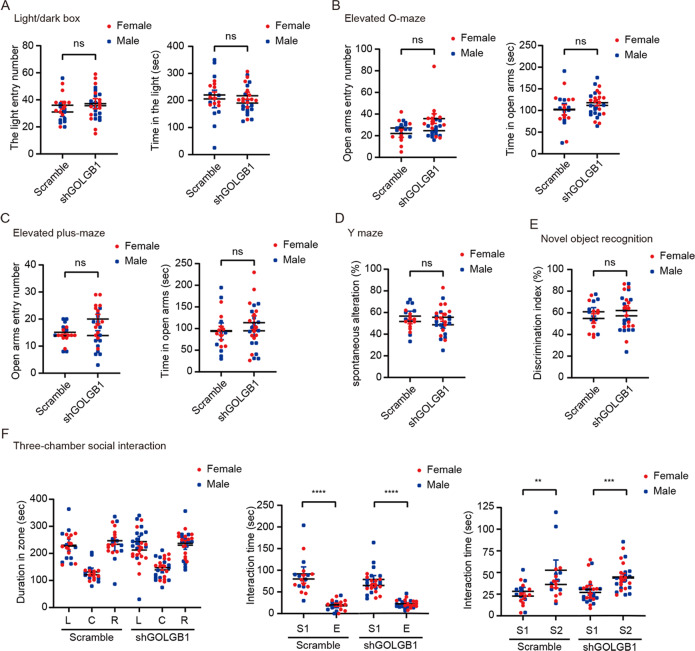
Downregulation of *Golgb1* has no effects on anxiety, memory, and social behaviors in mice. Mice of both sexes injected with AAVs expressing shGOLGB1 or a scrambled control shRNA were subjected to behavioral tests at 2 months of age. **A** In the light/dark box test, mice were studied for their time spent in the light box and entry numbers to the light box. (**B**, **C**) In the elevated O-maze test (**B**) and the elevated plus-maze test (**C**), mice were studied for their time spent in the open arms and open arm entry numbers. **D** In the Y-maze test, mice were studied for their spontaneous alternation percentage. **E** In the novel object recognition test, mice were studied for their discrimination index for the novel over familiar objects. **F** In the three-chamber social interaction test, mice were first studied for their duration in the left (L), central (C), and right (R) chambers during habituation (left panel). Mice were then tested for their time spent interacting with a strange mouse (S1) and with an empty cage (E) (middle panel). Finally, mice were tested for their time spent interacting with the familiar S1 mouse and with a new strange mouse (S2) (right panel). *n* = 27 (13 females and 14 males) for shGOLGB1 mice, *n* = 19 (ten females and nine males) for scrambled control mice. ns not significant; two-tailed Student’s *t*-test.

We also examined anxiety-like behaviors in *Golgb1* KD mice. In the open field test, although *Golgb1* KD mice exhibited increased numbers of center entries, their time spent in the center square was not different from that of controls (Fig. 3A). In the light/dark box test, both *Golgb1* KD and control mice showed comparable time spent in the light box and similar light box entry numbers (Fig. 4A). In the elevated O-maze test (Fig. 4B) and the elevated plus-maze test (Fig. 4C), both *Golgb1* KD and control mice had comparable time spent in the open arm and similar open arm entry numbers. Together, these results suggest that *Golgb1* downregulation has no effect on anxiety-like behaviors in mice.

In the Y-maze test, *Golgb1* KD mice showed no differences in their spontaneous alternation percentage compared to controls (Fig. 4D). In the novel object recognition test, *Golgb1* KD mice also showed no differences in their discrimination on the novel and familiar objects compared to controls (Fig. 4E). In the three-chamber social interaction test, *Golgb1* KD mice showed no differences in their social interaction behaviors compared to control mice (Fig. 4F). These results suggest that *Golgb1* downregulation does not impair memory and social behaviors in mice.

**Fig. 5:**
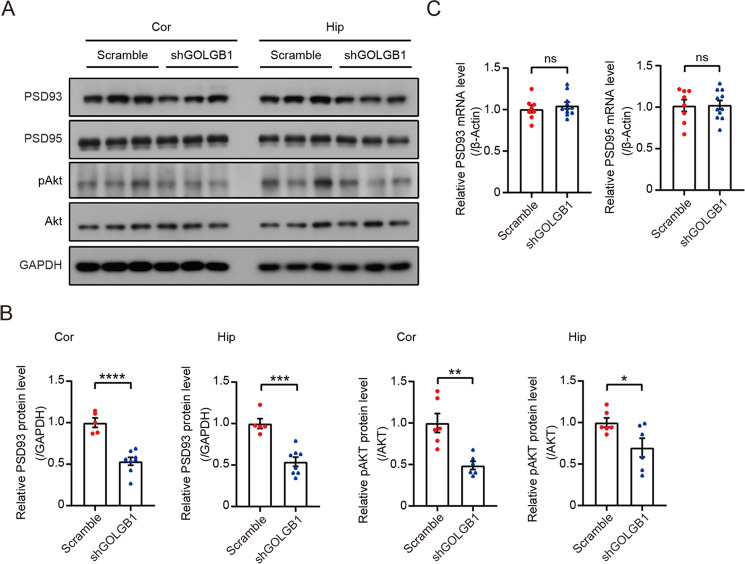
Downregulation of *Golgb1* reduces PSD93 protein levels and Akt phosphorylation. **A**, **B** Cortical (Cor) and hippocampal (Hip) tissues from mice injected with AAVs expressing shGOLGB1 or a scrambled control shRNA were collected. Equal amounts of protein lysates were subjected to western blot to study indicated proteins (**A**). Protein levels of PSD93 were quantified and normalized to those of GAPDH for comparison. *n* = 8 for shGOLGB1 mice, *n* = 5 for scrambled control mice. Protein levels of phosphorylated Akt (pAkt) were quantified and normalized to those of Akt for comparison. *n* = 6 for each group (**B**). *p < 0.05, **p < 0.01, ***p < 0.001, ****p < 0.0001; two-tailed Student’s *t*-test. **C** RNAs were extracted from cortical and hippocampal tissues of treated mice. The mRNA levels of PSD93 and PSD95 were determined by qRT-PCR and normalized to those of β-actin for comparison. *n* = 11 for shGOLGB1 mice, *n* = 8 for scrambled control mice. ns: not significant, two-tailed Student’s *t*-test.

Finally, to explore the potential molecular mechanism underlying BD pathogenesis caused by GOLGB1 deficiency, we checked several proteins whose alternations have been linked to BD. The results showed that *Golgb1* downregulation resulted in reductions in PSD93 and Akt phosphorylation protein levels in the cortex and hippocampus (Fig. 5A, B). However, *Golgb1* downregulation had no effect on mRNA levels of PSD93 and PSD95 (Fig. 5C), suggesting that PSD93 protein reduction upon *Golgb1* downregulation is not attributed to its gene expression alternation.

## Discussion

BD has been demonstrated to have a strong heritability [1, 18, 19]. However, although multiple genes have been identified as susceptibility loci for BD, they only account for a portion of BD occurrence. Additional studies in affected pedigrees shall help identify new disease-causing mutations and provide new insight into disease mechanisms.

In the present study, we carried out WGS for a Chinese BD family that shows an autosomal dominant mode of inheritance. From filtered variants, we identified 23 SNVs and three INDELs as candidate causal variations, as they were potentially malignant and heterozygous in all affected members but not mutated in unaffected members within this family. Among the 26 genes carrying SNVs or INDELS potentially associated with BD, we found that *GOLGB1*, *NEU1*, and *TBC1D16* were previously linked to BD to some extent.

The *GOLGB1* gene encodes GOLGB1/Giantin, a protein belonging to the golgin family that resides and modulates the vesicle trafficking network within the Golgi stack [11]. The exact function of GOLGB1 remains largely unclear, though several studies found that GOLGB1 could regulate protein glycosylation [12] and modulate ciliogenesis by controlling dynein-2 localization [13, 14]. In addition, homozygous loss of function mutation of *Golgb1* leads to several osteochondrodysplasia and late embryonic lethality in rats but only cleft palate in mice [12, 15], suggesting that GOLGB1 may regulate osteogenesis and/or chondrogenesis. Herein, we identified a frameshift INDEL in the *GOLGB1* gene in this Chinese BD family. Previously a *GOLGB1* missense mutation was also identified in a Caucasian BD family [4]. Therefore, we further studied the potential contribution of GOLGB1 deficiency to BD. We did not identify the two mutations or any other mutations in exons 9 and 15 of the *GOLGB1* gene in 182 sporadic BD patients and 146 controls, implying that BD-associated *GOLGB1* mutations may be rare. We then used AAV infection to downregulate *Golgb1* expression in the mouse brain. Although mice with *Golgb1* downregulation were morphologically normal, they exhibited elevated locomotor activity in the open field test and anti-depressive activities in the tail suspension test and the 2-bottle choice sucrose preference test, but depressive behavior in the forced swim test. These behaviors resemble the dual character of BD patients with both mania and depression.

PSD93 is a scaffold protein in the post-synaptic density of excitatory neurons and regulates synaptic plasticity. Mutations affecting *DLG2*, the gene encoding PSD93, have been associated with a series of neurodevelopmental psychiatric disorders, including schizophrenia and potentially BD [20, 21]. Dysregulation of the PI3K/Akt signaling pathway has been found and proposed as an important cause of BD [22]. Here we found that downregulation of *Golgb1* in the brain also reduced PSD93 protein levels and Akt phosphorylation. Therefore, our results suggest that GOLGB1 deficiency may cause the occurrence of certain BD phenotypes, possibly through altering multiple pathways such as PSD93 and PI3K/Akt signaling.

Interestingly, we also identified candidate causal variations in another two Golgi-related genes, *GOLIM4* and *COG6*. GOLIM4 encodes Golgi integral membrane protein 4 (GOLIM4) that may function in protein cargo transport through the Golgi apparatus and endosome-Golgi retrieval [23, 24]. The COG6 protein encoded by *COG6* is a member of the conserved oligomeric Golgi (COG) complex that plays an important role in Golgi trafficking and glycosylation enzyme positioning [25]. Biallelic mutations of *COG6* lead to congenital disorders of glycosylation, with features such as liver abnormality, microcephaly, and developmental disability [25]. Identification of candidate causal variations in multiple Golgi-related genes raises a possibility that compromised Golgi functions contribute to BD pathogenesis; and this deserves further scrutiny.

The *NEU1* gene encodes neuraminidase 1 (NEU1) that cleaves terminal sialic acid residues on glycoproteins and glycolipids. Mutations in *NEU1* lead to sialidosis, a lysosomal storage disease that can either occur at an early age with marked severity (dysmorphic type) or be late-onset with mild phenotypes (cherry red spot-myoclonus syndrome or normosomatic type). NEU1 was found to secret into exosomes upon inflammatory stimulus and exovesicular NEU1 was found to clear cell surface polysialic acid rapidly and thus lead to BDNF release [16, 26]. Since BDNF has been associated with BD [27–29], NEU1 alternation could potentially participate in BD as well.

The TBC1D16 protein encoded by *TCB1D16* is a member of the Tre2/Bub2/Cdc16 (TBC) domain-containing family proteins. TBC1D16 has GTPase activator activity and is involved in receptor recycling regulation [30]. One study found that BD patients with a history of suicidal behavior had decreased overall methylation in intron 3 of the *TBC1D16* gene compared to controls, though whether and how *TBC1D16* expression is altered in BD is unknown [17].

In summary, herein, we have identified several candidate causal variations in a Chinese BD family. Through combining functional investigation, we have demonstrated that *GOLGB1* is a strong BD risk gene candidate whose deficiency may result in BD phenotypes possibly by affecting PSD93 and PI3K/Akt signaling. Further corroboration in large patient cohorts and additional functional studies shall help conclude the causality of *GOLGB1* and other risk gene variations identified in this family.

## Materials and methods

### Human samples

Three BD patients who met the ICD-10 criteria of bipolar disorders in a Chinese family, including the grandfather, the father, and the daughter were diagnosed by two senior psychiatrists, and treated at Xiamen City Xianyue Hospital (Fig. 1). Other family members recruited in this study, including the mother, the uncle, and the grandmother were reportedly healthy. Details of the clinical features of the three affected members used in this study are compiled in Supplementary Table 1. Moreover, 182 sporadic BD patients and 146 healthy controls were recruited and tested for *GOLGB1* mutations. This study was approved by the Medical Ethics Committee of Xiamen City Xianyue Hospital. Informed consent was obtained from participants.

### Whole genome sequencing (WGS)

WGS was carried out at Novogene Bioinformatics Technology Co., Ltd (Beijing, China). Briefly, genomic DNA was extracted from peripheral blood and fragmented to an average size of ~350 bp. DNA library was created using established Illumina paired-end protocols and subjected to WGS using the Illumina Novaseq 6000 platform (Illumina Inc., San Diego, CA, USA) to generate 150-bp paired-end reads with a minimum coverage of 10× for ~98.5% of the genome (average sequencing depth over 30×).

### WGS data analysis

After sequencing, raw reads were filtered by in-house quality control software to remove low-quality reads. Clean reads were aligned to the reference human genome (hs37d5) using the Burrows–Wheeler Aligner [31], and duplicate reads were marked using sambamba tools [32].

SNVs and INDELs were called with samtools to generate gVCF [33]. The raw calls of SNVs and INDELs were further filtered with the following inclusion thresholds: (1) read depth >4; (2) root mean square mapping quality of covering reads >30; and (3) the variant quality score >20.

Annotation was performed using ANNOVAR (2017June8) [34]. Annotations included minor allele frequencies from public control data sets as well as deleteriousness and conservation scores, enabling further filtering and assessment of the likely pathogenicity of variations.

To filter rare variations, we first selected variations with a MAF less than 0.01 in 1000 genomic data (1000g_all) [35], esp6500siv2_all [9], gnomAD data (gnomAD_ALL and gnomAD_EAS) [10], and in-house Novo-Zhonghua exome database from Novogene. After discarding synonymous SNVs and small fragment nonframeshift (<10 bp) INDELs in the repeat region defined by RepeatMasker, we analyzed only nonsynonymous SNVs and INDELs occurring in exons or splice sites (splicing junction 10 bp). Variations were screened according to scores of SIFT [36], Polyphen [37], MutationTaster [38], and CADD [39] softwares. Potentially deleterious variations were reserved if the scores of more than half of these four softwares support the harmfulness of variations [40]. To better predict the harmfulness of variation, the American College of Medical Genetics and Genomics (ACMG) classification system was also used, which classifies variations into pathogenic, likely pathogenic, uncertain significance, likely benign, and benign [41].

To identify loci linked to BD, we performed an independent genome-wide scan for linkage in this family. This linkage analysis using merlin tools and the Perl, combined with the family high throughput sequencing data and the HapMap database of Chinese population (CHB) allele frequency, using the known SNP as a marker linkage analysis to get the chain candidate area.

The relationship between proband and parents was estimated using the pairwise identity-by-descent (IBD) calculation in PLINK [42]. The IBD sharing between the proband and parents in all trios is between 45 and 55%.

Given the inheritance pattern of the pedigree, dominant genetic modifiers seem to be responsible for disease pathogenesis in this family. Therefore, from filtered rare variations, we identified variations that were heterozygous in all three patients but not mutated in unaffected family members as candidate causal variations.

### *GOLGB1* sequencing

Genomic DNAs were extracted from BD patients and controls and used as PCR templates to amplify exons 9 and 15 of the *GOLGB1* gene (NM_004487). Primer pairs used were: Exon9_forward (5′-AGAAGGGCTTTCTCTCTAGCATA-3′) and Exon9_reverse (5′-TGGTTCAATTGGTTTGAGTACAGAT-3′), and Exon15_forward (5′-GGAAGAAACTGGGTGAAGGGTA-3′) and Exon15_reverse (5′-GTGGACTGTTAGGTGCTGGTTTC-3′). PCR products were then subjected to Sanger sequencing.

### Mice

C57BL/6J wild-type mice were housed under Specific-Pathogen-Free conditions at Xiamen University Laboratory Animal Center. Mice were kept on an 8:00–20:00 light/dark cycle with free access to phytoestrogen-free chow and water. Animal procedures were carried out in accordance with the guidelines of the National Institutes of Health Guide for the Care and Use of Laboratory Animals and were approved by the Animal Ethics Committee of Xiamen University.

### Mouse primary neuron culture

Primary neurons were derived from postnatal day 0 (P0) mouse brains as described previously [43]. Briefly, hippocampal and cortical tissues were removed from the P0 mouse brain and then dissociated with 0.25% trypsin and DNase I (0.2 kU/mL, Worthington) at 37 °C for 15 min. Neurons were cultured in a neurobasal medium (Gibco) supplemented with 2% B27 (Thermo Fisher Scientific).

### Adeno-associated virus (AAV) infection

AAV2/9 (serotype 2/9) viruses carrying mouse *Golgb1* shRNAs or scrambled control shRNA were packaged by OBIO Technology (Shanghai, China). There were three *Golgb1* shRNAs used: shGOLGB1 #1: 5′-GAGGAGAAAGCTGGAGGAA-3′, shGOLGB1 #2: 5′-ACTGCCATGGAATCGAATAAT-3′, and shGOLGB1 #3: 5′-GTTTCACGGGTCACCTATAAA-3′. The scrambled control shRNA sequence is: 5′-CCTAAGGTTAAGTCGCCCTCG-3′. Cultured mouse primary neurons were infected with AAV for 3 days in vitro (DIV) and analyzed at 10 DIV.

For in vivo injection, one microliter of AAV containing shGOLGB1 #2 or scrambled control shRNA (5 × 10^12^ V.G./ml) was slowly injected into lateral ventricles (2 mm distance from ventral to skin and 2/5 from lambda suture to the eye) of P0 C57BL/6J mice under hypothermic anesthesia. After injection, mice were put on a warming pad for body temperature recovery.

### Animal behavioral tests

Treated mice at 2 months of age, including ten female and nine male mice injected with AAVs expressing scrambled control shRNA and 13 female and 14 male mice injected with AAVs expressing shGOLGB1 #2 were subjected to various behavioral tests. All behavioral analyses were carried out in a double-blinded manner. Habituation was done in the testing room for more than 30 min at the beginning of each test day. All tests were carried out by researchers blinded to mouse genotype. Data were recorded and analyzed using the Smart 3.0 video tracking system (Panlab, Harvard Apparatus). Procedures for the open field test, the tail suspension test, the elevated plus-maze test, the three-chamber social interaction test, the Y-maze test, and the novel object recognition test were reported previously [43, 44]. Procedures for other behavioral tests were as the following.

For the two-bottle choice sucrose preference test, mice were first acclimatized to two identical bottles, one filled with water and the other one filled with water containing 1% sucrose, for 36 h. Mice were then fasted overnight without food and water for 12 h. After fasting, mice were presented with water and 1% sucrose again. The two bottles were weighed and exchanged positions every 12 h three times. Sucrose preference was determined as the ratio of total sucrose to water consumption.

For the forced swim test, mice were placed in the water with a temperature of 25 °C in a cylinder (21 cm in diameter and 30 cm in height) for 7 min. Total immobility duration during the last 6 min was recorded for comparison.

The dark/light box consisted of one black/dark (15 cm^3^ × 20 cm^3^ × 25 cm^3^) and one light (30 cm^3^ × 20 cm^3^ × 25 cm^3^) plexiglass compartment that were connected by a tunnel. Mice were placed into the light box and allowed to move freely for 10 min. The time spent in the light box and the number of entries into the light box were recorded for comparison.

Elevated O-maze consisted of an elevated circular platform with two opposite quadrants enclosed and two open. Animals were placed in the center of one open arm and let explore open and closed arms for 5 min. The time spent in open arms and numbers of open arm entries were analyzed.

### Western blot

Samples were lysed in TEN buffer containing 50 mM Tris-HCl, pH 8.0, 150 mM NaCl, 2 mM EDTA, and 1% NP-40, supplemented with protease inhibitors and phosphatase inhibitors. Protein concentration was determined by BCA assay (BCA Protein Assay Kit, Thermo Fisher Scientific). Equal amounts of protein samples were subjected to SDS-polyacrylamide gel electrophoresis and PVDF membrane transfer. Proteins were identified by incubating with indicated primary antibodies and then with appropriate HRP-conjugated secondary antibodies. Protein band intensities were determined using the Image J software [45]. Antibodies used were: anti-GAPDH (Abways, ab0037), anti-PSD93 (Abcam, ab151721), anti-PSD95 (Cell Signaling Technology, 3450 S), anti-Akt (Cell Signaling Technology, 9272 S), anti-phosphorylated Akt (Cell Signaling Technology, 9271 S), and HRP-conjugated secondary antibodies (Thermo Fisher Scientific, 31430 and 31460).

### Quantitative real-time PCR (qRT-PCR)

Total RNAs were isolated using TRIzol reagent (Life Technologies). After reverse-transcription using Superscript III transcriptase (Invitrogen), samples were analyzed on a LightCycler® 480 Real-Time PCR System (Roche Applied Science, Basel, Switzerland). PCR primers used are the following:

mGOLGB1-F, 5′-GCCTTCACTAAGAGCATGTCAT-3′;

mGOLGB1-R, 5′-GCTGATCCTTTAGAGCAATGCAG-3′;

mPSD93-F, 5′-AAACGCTCCCTGTATGTCAGA-3′;

mPSD93-R, 5’-CCCCATCTAGTGTGACCCTTC-3’;

mPSD95-F, 5′-TGAGATCAGTCATAGCAGCTACT-3′;

mPSD95-R, 5′-CTTCCTCCCCTAGCAGGTCC-3′;

mActin-F, 5′-GGCTGTATTCCCCTCCATCG-3′;

mActin-R, 5′-CCAGTTGGTAACAATGCCATGT-3′.

### Immunostaining

Brain samples of mice were fixed in 4% paraformaldehyde, sequentially dehydrated in 20, 25, and 30% sucrose solution, frozen in OCT compound, and then prepared as 15-μm slices. Slices were incubated with indicated primary antibodies overnight at 4 °C, followed by incubation with appropriate secondary antibodies conjugated with fluorescence and DAPI for 60 min at room temperature. The fluorescence microscope images were acquired by an A1R (Nikon) confocal microscope. Antibodies used were: anti-NeuN (Cell Signaling Technology, 94403 S), anti-GFAP (Proteintech, 16825-1-AP), anti-Iba1 (Wako, 019–19741), and Alexa fluor 594-conjugated goat anti-rabbit IgG (H C L) secondary antibody (Thermo Fisher Scientific, A-11012).

### Statistical analyses

Statistical analyses were performed using GraphPad Prism 8.3 software (GraphPad Software). Sample sizes were determined based on the assumption of a normal distribution and similar variability between experimental groups. No animals or samples were excluded from or randomized in the analyses. The normality distribution was corroborated using the Kolmogorov–Smirnov test. Two-tailed Student’s *t*-test was used for the comparison of two independent groups. The variances were similar between groups. Data represent mean ± standard error of the mean (SEM). *p* < 0.05 was considered to be statistically significant.

## Supplementary information

Supplementary Table 1

## Data Availability

The data sets generated in this study are available from the corresponding author upon reasonable request.
