# Supplementary material for: Pedigree-based study to identify GOLGB1 as a risk gene for bipolar disorder
Source: Transl Psychiatry. 2022 Sep 17;12:390. doi: 10.1038/s41398-022-02163-x (PMC9482626; doi:10.1038/s41398-022-02163-x)
Supplement: Supplementary file 1 — Supplementary Table 1 [file 41398_2022_2163_MOESM1_ESM.docx]

| **Supplementary Table 1. Clinical features of the three affected family members.** | | | |
| --- | --- | --- | --- |
|  | Grandfather | Father | Daughter |
| Age of onset, years | 43 | 32 | 18 |
| Predisposing factors | No | No | Stressful life events |
| Episode at first diagnosis | Depression | Depression | Hypomania |
| Predominant symptom | Depression | Depression | Depression |
| Months in hypomania episode following initial depression | 169 | 6 | 0 |
| Bipolar disorder (BD) subtype | BD, currently in remission | BD, currently in remission | BD, currently in remission |
| Lifetime suicide attempt | 0 | 0 | 0 |
| Current antidepressant drugs | Bupropion | / | Escitalopram |
| History of antidepressant drugs | Doxepin, fluoxetine, duloxetine, venlafaxine, paroxetine | Fluoxetine | / |
| Current mood stabilizers | Lamotrigine | Taking lithium irregularly | Lurasidone |
| History of mood stabilizers | Lithium, sodium valproate | / | Lithium |
| Psychosocial function | Normal life | Mild impairment in work | Normal learning |
